# The Use of Intergroup Social Comparison in Promoting Water Conservation: Evidence from a Survey Experiment in China

**DOI:** 10.3390/ijerph19137749

**Published:** 2022-06-24

**Authors:** Yijie Wang, Lei Xie, Shuang Li

**Affiliations:** 1 Institute of Governance, Shandong University, Qingdao 266237, China; yijiewang@sdu.edu.cn (Y.W.); lei.xie@sdu.edu.cn (L.X.); 2 School of Politics and Public Administration, Shandong University, Qingdao 266237, China

**Keywords:** intergroup social comparison, water conservation, prior water usage, strength of comparison

## Abstract

This study examines the effect of the intergroup social comparison approach in promoting water conservation. In an online survey experiment, participants first encountered normative persuasive messages, informative persuasive messages, or intergroup social comparison messages and then reported their willingness to conserve water, prior water usage, and demographic information. Results showed a significantly higher willingness to conserve water in the intergroup social comparison condition compared to other conditions. We further investigated whether this social comparison effect was moderated by prior water usage and the strength of comparison. Results suggest that prior water usage, rather than the strength of comparison, moderated the influence of intergroup social comparison. Moreover, the moderating effect of prior water usage only works when participants receive a low level of strength of comparison.

## 1. Introduction

Water scarcity and stress are great challenges all around the world. The World Resource Institute has estimated that 17 countries that are home to a quarter of the world‘s population face extremely high levels of water stress, where more than 80% of their available supply, on average, is withdrawn to irrigate agriculture, industries, and municipalities every year [1]. By 2025, two-thirds of the world’s population could be under water stress conditions [2]. In regions that experience water scarcity, the impacts can be understated but far-reaching in posing economic development obstacles and driving social instability concerns. The World Economic Forum declared that the water crisis was the top global risk in terms of impact [3].

In light of these challenges, finding strategies to achieve sustainable levels of water consumption is becoming an imminent issue in both academic research and the public policy agenda. In efforts to secure water supply into the future, demand-side management has emerged as an important complement to the traditional supply-side approaches that increase available water via technical improvements (e.g., desalinization, rainwater harvesting, wastewater recycling, water-saving appliances) [4]. Different from supply-side management, demand-side management focuses on the amount and efficiency of water use by consumers [5,6]. The World Health Organization suggests that 50–100 L of water per person per day is sufficient for personal and domestic uses, including washing clothes, personal and household hygiene, and other activities. Yet the domestic consumption rates of many countries far exceed this amount. For example, people in the United States consume 372 liters of water on average per day [7], and the average domestic water consumption in China varies from 134 liters per person per day in urban areas to 100 liters per person per day in rural areas [8]. It is, therefore, necessary to manage water demand by encouraging individuals to conserve water in everyday lives to maintain sustainable water consumption.

Individual water conservation has been targeted using a range of intervention strategies (see [9] for a review). While it has been found that traditional intervention approaches such as pricing structures and prompts have limited impacts [10], recent developments in behavioral science have uncovered more potentially effective intervention strategies for promoting water conservation (see [10,11] for reviews).

### 1.1. Social Comparison

Social comparison, which informs individuals of their consumption relative to others, is an important behavioral-based strategy to foster water conservation. Environmental behaviors suffer from the working assumption that most people lack awareness and understanding of how their everyday behaviors affect the environment. Researchers have pointed out that feedback transmitting information about the effects of the behaviors of an individual or a group is effective in reducing their negative impacts on the environment [12]. While simple feedback is generally associated with small behavioral changes, feedback paired with a meaningful comparison can be more powerful [13]. According to the social comparison theory, individuals have a tendency to compare themselves to others (e.g., in terms of opinions or behaviors) in order to validate their own cognition and behaviors [14]. This theory indicates that cognition about one’s performance compared with others can elicit strong behavioral intentions [12]. The social comparison approach has been proven effective in boosting pro-environmental behaviors, such as energy conservation [15,16,17] and recycling [18,19].

However, only a small number of studies have been conducted in the area of water conservation [20,21]. Among them, a representative field experiment conducted in Atlanta carried out an information campaign by sending letters, including social comparison messages, to consumers during a severe drought. Several studies analyzed the data collected in this experiment and showed that the social comparison approach effectively reduced water consumption [22,23,24,25]. To be specific, Ferraro and colleagues [24] found that consumers who received the social comparison messages consumed 4.8% less water in the next months than consumers who were in the control condition. Moreover, Ferraro and Price [25] found that the social comparison message had a greater influence on water conservation than the mere pro-social information, which encouraged customers to conserve water, and the technical information, which introduced how individuals could conserve water. Experiments conducted in other regions have also provided evidence that confirms the effects of the social comparison approach, which informs customers of their consumption relative to their neighbors, in reducing water consumption [26,27,28].

Most of the related studies benefited from recent advances in water-measuring infrastructure and, therefore, could provide participants with detailed social comparison information about their own and the reference group’s daily water consumption. In other words, these studies elicited social comparison by providing each participant with tailored messages, which require water consumption data on the individual level. However, individual-level information is oftentimes costly and difficult to collect. Moreover, the tailored comparison information heavily restricts the application of the social comparison approach in mass environmental campaigns, such as presenting slogans in public places and launching media pro-environmental advertisements. In response to these problems, the present research investigates how social comparison messages given on the collective level instead of the individual level, which is defined as an intergroup social comparison (reviewed in detail in the next section), can be applied to encourage behavioral intention towards water conservation.

### 1.2. Intergroup Social Comparison

According to the characteristics of the counterpart in comparison, there are two types of social comparisons: intragroup comparison and intergroup comparison [29].

Intragroup comparison refers to the process in which individuals compare themselves to their ingroup members [30,31], which motivates behavioral change by providing an ingroup norm to follow [32]. By enhancing the salience of discrepancies between personal behavior and the ingroup norm, this type of comparison drives individuals to evaluate their abilities and encourages them to reduce any observed discrepancies. Furthermore, Festinger [14] stated that people have a tendency to compare themselves to those who are similar to them. People who are similar or comparable are perceived to be more likeable and more persuasive [33]. Thus, the intragroup comparison, providing information about the performance of people with similar characteristics in a larger ingroup (such as households living in the same neighborhood), has been found to be an effective behavioral-based strategy to affect people’s willingness to engage in water conservation [22,23,27,28].

The other type of social comparison is intergroup comparison, in which people compare their own group to an outgroup [30,34]. According to the social identity theory [35,36], intergroup comparison can make the distinctiveness among ingroup and outgroup members salient and further elicit competition among the groups. Rabinovich and colleagues [37] have successfully applied the intergroup comparison approach to promoting sustainable behaviors. In their work, British participants were asked to write down the ways that being British differed from being Swedish or from being American. Results showed that those who compared themselves to Americans were more willing to engage in pro-environmental behaviors than those who compared themselves to Swedish citizens. In another study, Ferguson and colleagues [29] asked university students to compare themselves with past or future generations of students and then report their willingness to perform sustainable behaviors, and found that students who compared themselves to past generations reported more willingness to perform sustainable behaviors than those who compared themselves to future generations. Results from these studies indicated that intergroup comparison is a promising approach to motivating sustainable behaviors. However, water conservation behavior is not explicitly targeted in these studies, and thus, the effect of intergroup comparison on water conservation has yet to be investigated. Furthermore, in previous studies, social comparison is strongly based on participants’ subjective evaluations rather than objective information. In the present research, we investigate how the intergroup comparison approach, which is based on objective data instead of subjective expectations, can be applied to elicit effective behavioral change in reducing residential water consumption.

### 1.3. Moderators in Social Comparison Effect

Previous studies have found that the social comparison effect appears to be stronger among high-consuming households than low-consuming households in both water and energy domains [25,26,38,39]. For example, Ferraro and Price [25] found that social comparison messages reduced water consumption by 5.28% among high-consuming households and 2.72% among low-consuming households compared to households in the control condition. In other studies, the social comparison approach resulted in a non-significant effect among low-consuming households, sometimes even a boomerang effect of increasing water consumption [32,40]. These previous studies using the intragroup comparison approach seem to indicate that individuals’ prior water usage moderates the social comparison effect. However, one common limitation shared by these studies is that they are unable to untangle whether the influence of the social comparison approach on water conservation is actually moderated by individuals’ prior water usage or by the strength of comparison since studies using the intragroup comparison could elicit a high degree of correlation between prior water consumption and the strength of comparison. In such studies, the strength of comparison depends on the distance between an individual’s performance (i.e., prior water usage) and the performance of the relevant comparison group [27]. Therefore, the more water one consumes, the larger the distance between the performances. For example, when the high-consuming households are informed that they are badly underperforming relative to their peers, they naturally receive stronger comparison manipulation.

However, intergroup comparison based on collective-level information can clearly distinguish one’s prior water usage from the strength of comparison since the strength of comparison is measured by the distance between the ingroup performance and the outgroup performance, where individuals’ own prior water usage is not directly involved. Therefore, intergroup comparison messages, which present individuals with the collective performance of their ingroups instead of their own performances, naturally decouple the strength of comparison from prior water usage on the individual level. In the current research, we present intergroup comparison information based on provincial data to investigate the moderating effect of prior water usage and the moderating effect of strength of comparison, respectively.

### 1.4. The Present Research

The present research examines how intergroup comparison can influence the willingness to engage in water conservation behaviors and whether prior water usage and the strength of comparison can moderate the social comparison effect. Similar to Brent and colleagues [27] and Komatsu and Nishio [41], we did not use a no-information condition as the control group since we aimed to compare the effects of different types of persuasive messages instead of investigating whether a certain type of message works better than no message at all. Specifically, we used a between-subject design with three experimental conditions (i.e., the normative persuasive condition, the informative persuasive condition, and the intergroup social comparison condition), of which the normative persuasive condition (i.e., presenting participants with messages merely persuading them to conserve water) served as the primary control group and the informative persuasive condition (i.e., presenting participants with messages introducing the local water scarcity and persuading them to conserve water) served as the secondary control group.

To generate the intergroup social comparison, we employed the most commonly recognized group identity: place of residence. Shandong Province was chosen from all 34 provinces in China as the research group because it is a water-scarce province (ranked 26th among all 34 provinces) and, thus, has an appropriate discrepancy from other provinces. Another reason to choose Shandong Province is that it will be convenient for the researchers to conduct replicational field experiments in the future.

A survey experiment was conducted on Credamo, which is a professional research platform with a sample database of more than 1.5 million Chinese participants. It can provide large-scale data collection services and has been recognized by journals in the fields of psychology, management, sociology, and environmental science. Participants who reside in Shandong Province were recruited. They were provided with either normative persuasive messages, informative persuasive messages, or intergroup comparison messages (i.e., the rank of the water resources of Shandong Province among all provinces in China). They were then asked to report their willingness to perform water conversation behaviors.

Based on the theoretical framework discussed in the introduction, we hypothesized that social comparison messages would cause a higher willingness to conserve water relative to normative persuasive messages and informative persuasive messages. We also predicted that the effect of intergroup comparison on willingness to conserve water would be moderated by individuals’ prior water usage, such that participants who conserve water to a higher degree would show a lower willingness to reduce water consumption. Similarly, we hypothesized that the strength of comparison would moderate the effect, such that participants who receive a stronger level of comparison would show a higher willingness to conserve water. Finally, we investigate whether the moderating effect of prior water usage would operate differently under different levels of strength of comparison, that is, whether the two potential moderators interact with each other.

## 2. Method

### 2.1. Participants

The current research surveyed individuals who resided in Shandong Province, China, in January and February of 2021. Data were collected via Credamo, and 501 participants participated in this online survey experiment. Among these participants, 131 were ruled out because they did not pass the attention check, which indicates that they did not carefully read the experimental materials (We added 2 attention check items in the survey experiment to check whether participants carefully read the experimental materials. Data of those who gave correct answers to both items were included in the final analysis. An example of the attention check item is: ‘This item is to check if you are carefully doing the experiment. Please choose 5.’). Five participants were ruled out because their response time was too long (A long response time could decrease the reliability of the collected data. Considering the length of our survey questionnaire (M = 354.67, *SD* =127.78), we chose 900 s as the cutoff of outliers.). Finally, data from 365 participants were included in the statistical analysis. Among these 365 participants, 56.2% were female and 43.8% were male. The mean age of the participants was 27.55 years old (*SD* = 7.69).

### 2.2. Procedure

The current survey experiment was programmed into a questionnaire and activated on Credamo. Individuals registered on this platform received a notification that directed them to the questionnaire. By using the screening function, only individuals whose place of residence was Shandong Province were allowed to participate in our study in order to ensure that all participants shared the same group identity (residents in Shandong Province) when compared with outgroups.

Participants were randomly assigned to one of the three experimental conditions, in which they were presented with different persuasive messages (The messages used in the 3 experimental conditions can be found in Supplementary File S1). In the *normative persuasive* condition (*n* = 122), the participants read a generic statement about the scarcity of water resources, stating that everybody should conserve water. In the *informative persuasive* condition (*n* = 123), in addition to the message presented in the normative persuasive condition, participants were provided with precise statistical data indicating the scarcity of water resources in their hometown (i.e., Shandong Province). In the *intergroup comparison* condition (*n* = 120), the same statement as in the normative persuasive condition was first presented, and then participants were asked to estimate the rank of per capita water availability in their hometown province (i.e., Shandong Province) among all 34 provinces in China. Afterwards, participants were provided with the actual rank information (i.e., 26th) and asked to indicate whether their estimation was higher, lower, or consistent with the actual rank. After reading the persuasive messages, participants were asked to indicate their willingness to conserve water and their prior water usage and to answer some demographic questions.

### 2.3. Measures

*The intention to conserve water.* The dependent variable used in this study was the participants’ intentions to engage in water conservation. Participants indicated how likely they would engage in water-conservation behaviors (from 1 = extremely unlikely to 100 = extremely likely). The behaviors are: ‘Use no more than one cup of water at a time to brush your teeth’ and ‘Use basins when washing hands and vegetables in order to recycle the water for flushing the toilet’. These items were selected from a comprehensive review of websites, opinion polls, and other resources that advise the public on water conservation behaviors and were recognized as effective conservation tips that were unrestrained by contexts (e.g., it did not matter if individuals were able to install water-efficient devices or not). Participants’ intention to conserve water was indicated by the mean score of the two items (*α* = 0.613).

*Perceived prior water usage*. Perceived prior water usage was measured as the index of individuals’ actual prior water usage since people’s subjective evaluation highly depends on their objective water usage. In addition, compared to collecting data of participants’ actual prior water usage, perceived prior water usage can be more easily obtained by participants’ self-report. In our study, the following item was used to measure perceived prior water usage: ‘Please indicate which of the following descriptions most closely matches how you use water in your daily life’. Participants had to respond using a 5-point Likert scale, ranging from 1 (I used to waste water) to 5 (I used to conserve water).

*Perceived strength of comparison*. In the current study, we constructed the intergroup comparison by comparing the per capita water availability of Shandong Province with that of the other provinces in China. Since the strength of comparison is defined by the distance between the ingroup performance and the outgroup performance, in this study, it is the ranking of the per capita water availability of Shandong Province (i.e., 26th) among all 34 provinces in China. In this situation, the strength of comparison does not vary for our participants, all of whom reside in Shandong Province, which makes it impossible to examine the moderating effect of the strength of comparison without any variation. To solve this problem, we used the participants’ perceived strength of comparison instead, which varies according to the participants’ cognitive process. To be specific, participants holding different estimations about the rank of Shandong Province in China would perceive different degrees of strength when informed of the actual rank (i.e., 26th).

We designed a two-question structure to measure the perceived strength of comparison. To be specific, the first question asked participants to estimate the rank of per capita water availability in their hometown province (i.e., Shandong Province) among all 34 provinces in China. The second question asked participants to indicate whether their estimation was higher, lower, or consistent with the actual rank after participants were informed that Shandong Province actually ranked 26th in China. Consequently, the perceived strength of comparison was calculated by subtracting the actual rank number from the participants’ estimated rank number. Negative values indicate that participants assumed Shandong Province would rank above 26th and later realized that Shandong Province actually owns fewer water resources than they expected when informed of the actual rank. For negative values, the larger its absolute value, the stronger the perceived strength of comparison. For instance, participants who estimated Shandong Province would rank in the top position (i.e., holding the most water resources in China) received the strongest strength after being informed that the actual rank was 26th. Conversely, positive values indicate that the participants assumed Shandong Province would rank below 26th and later realized that Shandong Province actually owns more water resources than they expected when informed of the actual rank.

## 3. Results

### 3.1. Main Effects

Statistical analyses were conducted in Stata 17.0. First, to check whether the participants were randomly assigned to three conditions, a MANOVA was conducted on age, education, and economic status, with the condition as the independent variable. The descriptive results are presented in Table 1, *p*’s ≥ 0.138, *F* ≤ 1.99, indicating that the randomization was successful.

**Figure 1:**
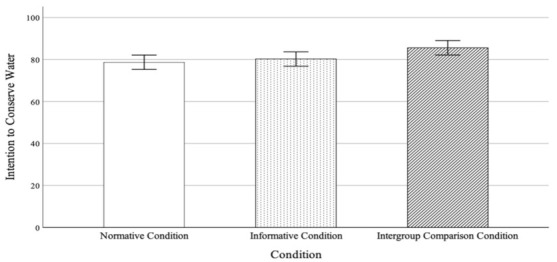
The main effect of the conditions on the intention to conserve water. The 90% confidence level was reported.

To test whether participants’ intention to conserve water differed among the conditions, an ANOVA with the condition as the independent variable and intention as the dependent variable was conducted. As shown in Figure 1, a significant main effect of the condition was observed, *F* (2, 362) = 3.03, *p* = 0.050, *η*^2^ = 0.016. The LSD post-hoc analysis revealed that intentions were significantly higher in the intergroup comparison condition (*M* = 85.62, *SD* = 17.88) compared to the normative persuasive condition (*M* = 78.72, *SD* = 26.03), *p* = 0.020, and marginally higher compared to the informative persuasive condition (*M* = 80.26, *SD* = 23.89), *p* = 0.069. These findings provide initial evidence for the effectiveness of the intergroup comparison approach in promoting water conservation. Since no significant difference between the normative persuasive condition and the informative persuasive condition (*p* = 0.600) was observed, to simplify the analysis, the normative persuasive condition was further used as the control group in the later moderating effect analysis. (We also did the moderator analysis with a full sample that included both control groups and found consistent results.)

### 3.2. Moderating Effects

#### 3.2.1. Prior Water Usage

**Figure 2:**
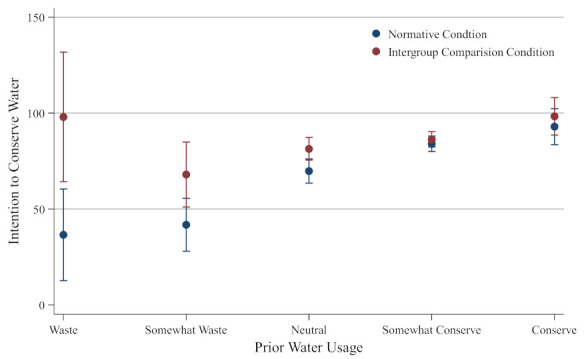
Intentions of participants with different prior water usage to conserve water. The 90% confidence level was reported.

Having demonstrated a significant main effect for the intergroup social comparison condition, our analyses proceeded to test the hypothesis about the moderating effect of prior water usage. To determine whether the effect of intergroup comparison on the willingness to conserve water was heterogeneous for respondents with different prior water usage, we conducted a regression with a planned interaction. Specifically, a dichotomous variable was computed, which was marked as 1 if participants were from the intergroup comparison condition and as 0 if participants were from the control condition (i.e., normative persuasive condition), and then a multiplicative term was computed as the product of prior water usage and the dichotomous variable. The moderating effect of prior water usage was then tested using regression, with the intention to conserve water as the dependent variable. Prior water usage was entered in the regression at the first step, followed by the dichotomous variable and the multiplicative term (see [42] for more details on this procedure). The results showed a significant effect of prior water usage (*b* = 15.304, *SE* = 2.375, *t* = 6.44, *p* < 0.001), a significant effect of the dichotomous variable (*b* = 39.746, *SE* = 13.430, *t* = 2.96, *p* = 0.003), and a significant interaction effect (*b* = −8.847, *SE* = 3.533, *t* = −2.50, *p* = 0.013). The marginal effects were plotted to further visualize how the effect of intergroup comparison varies under different levels of prior water usage. As shown in Figure 2, the intention to conserve water was significantly higher in the intergroup comparison condition than in the control condition among those who used to waste water, whereas as the degree of self-reported prior water conservation increases, the difference in intention to conserve water between the intergroup comparison condition and the control condition decreases. These results imply that the effect of intergroup comparison on water conservation is dependent on the individuals’ prior water usage. Compared to people who already conserve water, those who used to waste water are more responsive to intergroup comparison messages.

#### 3.2.2. The Strength of Comparison

Our second moderating analysis aimed to test the moderating role of the strength of intergroup comparison. The strength of intergroup comparison is measured by the difference between the estimated rank number and the actual rank number (i.e., 26th) of the per capita water resource in Shandong Province among all 34 provinces in China; thus, only participants in the intergroup comparison condition have scores in this specific measure. Therefore, we were unable to conduct a regression analysis, including the multiplicative term as the product of strength and condition, to analyze the interaction effect. Instead, we divided participants in the intergroup comparison condition into three subgroups (i.e., high-strength group, low-strength group, and reverse-treated group) and then conducted three separate linear regressions in each subgroup. The grouping rules are as follows: Those who assumed that Shandong Province holds rich water resources were assigned to the high-strength group; those who were less optimistic about the water resources of Shandong Province while still estimating that Shandong Province ranks above 26th were assigned to the low-strength group; those who estimated that Shandong Province ranks below 26th were assigned to the reverse group, since the later presented message indicating that Shandong Province owns more water resources than their expectation may have conversely influenced their water conservation intention. We used third as the cutoff value for the high- and low-strength groups. To be specific, those who estimated that Shandong Province ranked 1st to 3rd were included in the high-strength group, and those who estimated that Shandong Province ranked 4th to 25th were included in the low-strength group. We chose the third position as the cutoff value according to the following reasons: Firstly, the high-strength group needs to be large enough to be recognized, which makes it reasonable to choose the top ranking positions as the cutoff. Secondly, we tested several top ranking positions as the cutoff values (see Supplementary Tables S1 and S2) and obtained consistent findings. To simplify the results section, we only present the results analyzed using the third position as the cutoff value.

For each subgroup (i.e., high-strength group, low-strength group, reverse-treated group), we did a regression analysis on participants in this specific subgroup and participants in the control condition. Taking the high-strength group as an example, a dichotomous variable was computed, which was marked as 1 if the participants were from the high-strength group and as 0 if participants were from the control condition. The effect of intergroup comparison on the high-strength group was then tested using the regression with the dichotomous variable as the independent variable and the intention to conserve water as the dependent variable. For the high-strength group, the linear regression revealed a marginally significant effect of intergroup comparison on the intention to conserve water (*b* = 11.779, *SE* = 6.115, *t* = 1.93, *p* = 0.056). Similarly, for the low-strength group, the effect of intergroup comparison on the intention to conserve water was also significant (*b* = 6.379, *SE* = 3.202, *t* = 1.99, *p* = 0.048). No significant effect was found in the reverse group (*p* = 0.578), indicating that there was no boomerang effect of reverse intergroup comparison.

Although results showed a much larger coefficient value for the high-strength group (*b* = 11.779) than for the low-strength group (*b* = 6.379), it does not necessarily imply that the effect of the intergroup social comparison approach is moderated by the strength of comparison. To determine cross-model differences by examining the statistical significance of an effect within each model and numerically comparing the coefficients of the models has been criticized. In contrast, it is necessary to determine whether the difference between coefficients reaches a statistically significant level [43,44]. Following Mize and colleagues [45], we use Stata’s *suest* command to test the equality of effects across models. From the results shown in Table 2, the difference in the effect of the intergroup social comparison approach across two models under different strengths of comparison is not statistically significant (*p* = 0.132). These results indicate that the effect of intergroup comparison is significant, regardless of the strength of comparison.

#### 3.2.3. The Moderating Effect of Prior Water Usage under Different Levels of Strength of Comparison

We further investigated the moderating role of prior water usage under different levels of strength of comparison by separately testing the multiplicative term as the product of the condition and prior water usage among the high-strength, low-strength, and reverse-treated subgroups. (As in Section 3.2.2, we use 3 as the cutoff value when grouping participants into three subgroups. Consistent results were found when using other numbers as the cutoff values. The results are presented in Supplementary Tables S3 and S4.) For participants in the high-strength group and the control group, we conducted a regression analysis with the intention to conserve water as the dependent variable and included prior water usage as the dichotomous variable (0 = normative persuasive condition, 1 = high-strength intergroup comparison condition) and the interaction term as the product of prior water usage and the dichotomous variable. Results showed a significant effect of prior water usage (*b* = 15.304 *SE* = 2.557, *t* = 5.99, *p* < 0.001), but neither the effect of condition (*p* = 0.185) nor the effect of interaction was significant (*p* = 0.344). A similar regression analysis was performed for participants in the low-strength group and the control group. Results showed a significant effect of prior water usage (*b* = 15.304, *SE* = 2.388, *t* = 6.41, *p* < 0.001), a significant effect of the condition (*b* = 40.810, *SE* = 15.022, *t* = 2.72, *p* = 0.007), and a significant interaction effect (*b* = −9.266, *SE* = 3.955, *t* = −2.34, *p* = 0.020). Furthermore, a similar regression analysis was performed for participants in the reverse-treated group and the control group. Results showed a significant effect of prior water usage (*b* = 15.304, *SE* = 2.744, *t* = 5.58, *p* < 0.001), but neither the effect of the condition (*p* = 0.118) nor the effect of the interaction was significant (*p* = 0.143). The results of moderating effect of prior water usage under high-strength, low-strength, and reverse-treated group are presented in Table 3.

In summary, we found that the moderating effect of prior water usage was only concentrated on participants who received a low strength of comparison. In this situation, participants who used to waste water reported a higher intention to decrease their water consumption compared to those who used to conserve water. However, when participants received a high strength of comparison or realized that the water resources in Shandong Province were more plentiful than their estimation, the moderating effect of prior water usage disappeared.

## 4. Discussion

The current experiment focuses on the role of intergroup social comparison in affecting willingness to conserve water, along with several moderators discussed in prior research.

The results show that intergroup social comparison can significantly boost individuals’ willingness to conserve water. Generally, participants who received the intergroup comparison message reported more willingness to reduce residential water consumption than those who received the informative persuasive message or the normative persuasive message. This is in line with previous research that found the effectiveness of the intergroup social comparison approach in encouraging sustainable behaviors [29,37]. Compared with the studies targeting general sustainable behaviors, our study explicitly investigated the effect of intergroup comparison on water conservation. Our finding has meaningful implications for the design of water conservation campaigns. Compared with previous studies eliciting intragroup social comparisons by providing each participant with tailored messages [22,23,27,28], intergroup social comparisons given on the collective level have the advantage of reducing the cost of collecting individual information and widening the application scenarios from personal to public. On the basis of our findings, public environmental campaigns, such as pro-environmental slogans and mass media, are recommended to address the intergroup social comparison message. Communicating credible information about the better behavior and practices of outgroups, such as other cities or provinces, could elicit competition among the groups, resulting in increased water-saving efforts among local residents.

With regard to the moderating effects, a significant moderating effect of prior water usage and a non-significant effect of strength of comparison were shown. Moreover, the moderating effect of prior water usage operated differently under different levels of the strength of comparison. When the strength of comparison is small, the effect of intergroup comparison is concentrated among participants who perceive their prior water usage as wasteful. When the strength of comparison is large, the intergroup comparison approach works the same for participants who used to waste water and participants who used to conserve water. By distinguishing one’s prior water usage from the strength of comparison, the current study extends previous findings that only focused on the moderating effect of prior water usage [25,26,38,39]. Our findings regarding the effects of two moderators provide empirical evidence for policymakers and practitioners to improve campaign cost-effectiveness. Specifically, under conditions where the individuals’ perceived water usage is available, social comparison messages are recommended for targeting individuals who perceive themselves as wasting water since they are the most responsive subgroup. Under conditions where the individuals’ perceived water usage is unavailable, we recommend that practitioners provide intergroup comparison messages as strongly as possible by choosing the outgroup whose performance holds the largest discrepancy from the target group’s performance as the reference group.

Several limitations of the present research should be addressed. Firstly, we did not test the participants’ group identity with Shandong Province. Although they all currently reside in Shandong Province, their identification with Shandong Province might be different. According to the social identity theory, the social comparison approach should operate on the basis of salient group identities. To be specific, high-level (vs. low-level) group identification generally strengthens group-based effects. Therefore, the power of intergroup social comparison is supposed to be stronger for individuals who strongly identify themselves with the ingroup after activating the specific group identity (i.e., a local province in the present study). Future research could examine how the level of group identification moderates the effect of the social comparison approach, which can provide a better understanding of the underlying mechanisms through which the causal effect is generated. Moreover, it is also worth discussing which type of group identity is required to drive group members to conserve water. For example, Leach and colleagues [46] provided a hierarchical model distinguishing two types of group identification, which are self-investment and self-definition. Previous applications of the model [47,48] supported the assumption that self-investment and self-definition vary in their effects on group-related cognitions, emotions, and behaviors. Secondly, our study is prone to most of the threats to external validity that generally apply to survey experiments. Caution should be exercised when attempting to generalize these findings to other regions. We tested our hypotheses in a water-scarce province (ranked 26th out of 34 provinces) and found the effectiveness of intergroup social comparison in promoting water conservation and its boundary conditions. However, it is still unknown if this approach can be generalized to the water-sufficient provinces. Previous research has found a non-significant effect of the social comparison approach among low-consuming households and has sometimes even found a boomerang effect of increasing water consumption [32,40]. When this approach is applied to water-sufficient areas, a similar boomerang effect might be observed, which should be carefully examined in future research. Thirdly, consistent with previous studies using opt-in online samples [49], the participants were volunteers from a commercial online research panel, which may differ in important ways from a true probability sample of the study area. The limited representation of our sample suggests that the results of this study need to be replicated by additional experiments using samples of different characteristics, which would help consolidate or improve the findings of this study. Lastly, the current study used an online platform to inform people of the relevant information. Recent research has found that the methods of notification that inform people of sustainability achievements can influence their satisfaction [50]. Future research could examine how different notification methods, such as leaflets, emails, and SMS, affect the effectiveness of this approach.

## 5. Conclusions

To conclude, intergroup social comparison has a significant influence on people’s willingness to conserve water, and prior water usage rather than the strength of comparison moderates this intergroup social comparison effect. Moreover, the moderating effect of prior water usage only works when participants receive a low level of strength of comparison. Therefore, the current research emphasizes the importance of applying the intergroup social comparison approach in water conservation promotion and points out that increasing the strength of comparison is the most cost-effective way to obtain positive outcomes when policymakers and practitioners do not know the targets individually.

## Figures and Tables

**Table 1:** Mean scores and standard deviations on demographic variables.

Variable	Normative Persuasive Condition	Informative Persuasive Condition	Intergroup Comparison Condition
Age	27.27 ± 7.42	27.64 ± 7.57	27.73 ± 8.13
Education	5.52 ± 1.16	5.51 ± 0.96	5.54 ± 0.89
Economic status	3.09 ± 0.63	2.95 ± 0.57	2.98 ± 0.50

Education was reported on an 8-point scale, from 1 = lower than primary school to 8 = doctor. Economic status was reported on a 5-point scale, from 1 = far below average to 5 = far above average.

**Table 2:** Effect of intergroup comparison on high-strength and low-strength groups.

	Model 1	Model 2	Cross-Model Difference
	High-Strength	Low-Strength	
*B*	11.779 *	6.379 **	5.400
*SE*	(6.115)	(3.202)	(3.584)
*N*	141	207	
*R* ^2^	0.026	0.019	

* *p* < 0.10, ** *p* < 0.05.

**Table 3:** Moderating effect of prior water usage under high and low strength of comparison.

	High-Strength	Low-Strength	Reverse-Treated
*B*	−6.011	−9.266 **	−18.487
*SE*	(6.330)	(3.955)	(12.536)
*N*	141	207	138
*R^2^*	0.239	0.196	0.191

This table only reports results of the effect of the multiplicative term, which indicates whether the moderating effect of prior water usage exists. ** *p* < 0.05.

## Data Availability

The data that support the findings of this study are available from the corresponding author, [S.L.], upon reasonable request.

## Supplementary Materials

The following supporting information can be downloaded at: https://www.mdpi.com/article/10.3390/ijerph19137749/s1, File S1: Experimental Materials; Table S1: Effect of intergroup comparison under high and low strength of comparison. (Using different cutoff values as the grouping criteria); Table S2: Effect of intergroup comparison under high and low strength of comparison. (Using different cutoff values as the grouping criteria); Table S3: Moderating effect of prior water usage under different strength of comparison. (Using different cutoff values as the grouping criteria); Table S4: Moderating effect of prior water usage under different strength of comparison. (Using different cutoff values as the grouping criteria).
