# Supplementary material for: The Use of Intergroup Social Comparison in Promoting Water Conservation: Evidence from a Survey Experiment in China
Source: Int J Environ Res Public Health. 2022 Jun 24;19(13):7749. doi: 10.3390/ijerph19137749 (PMC9265829; doi:10.3390/ijerph19137749)
Supplement: Supplementary file 1 [file ijerph-19-07749-s001.zip › Supplementary File S1 - Experimental Materials.pdf]

International Journal of
Environmental Research and Public Health

MDPI

Supplementary File S1

Experimental Materials

The normative persuasive messages

Water is an essential requirement for life and health, and is not inexhaustible. Although most of the earth is covered by water, fresh water resources are extremely limited. We should cherish water resources and conserve water.

The informative persuasive messages

Water is an essential requirement for life and health, and is not inexhaustible. Although most of the earth is covered by water, fresh water resources are extremely limited. Shandong Province holds 1% of the national water resources, irrigates 6% of the national farmlands, produces 8% of the national grain, raises 7% of the national population, and supports 10% of the national economic aggregates. Despite being on the eastern coast and in the lower reaches of the Yellow River, Shandong Province is in fact one of the most severely water-scarce regions in China. We should cherish water resources and conserve water.

The intergroup social comparison messages

Water is an essential requirement for life and health, and is not inexhaustible. Although most of the earth is covered by water, fresh water resources are extremely limited. We should cherish water resources and conserve water.

Question 1: If all the 34 provinces in China are ranked from the top to the bottom in terms of water availability per person, which means the 1st-ranked province has the highest level of water resource per capita and the 34th-ranked province has the lowest level of water resouce per capita, what is your estimated rank of Shandong Province (from 1st-ranked to 34th-ranked)?

Question 2: According to the China Environmental Statistics Yearbook, Shandong Province ranks the 26th out of 34 provinces in terms of water availability per person. Is the correct answer higher than, lower than, or consistent to your estimation in the above question?
