# Supplementary material for: The Use of Intergroup Social Comparison in Promoting Water Conservation: Evidence from a Survey Experiment in China
Source: Int J Environ Res Public Health. 2022 Jun 24;19(13):7749. doi: 10.3390/ijerph19137749 (PMC9265829; doi:10.3390/ijerph19137749)
Supplement: Supplementary file 1 [file ijerph-19-07749-s001.zip › Supplementary Tables S1-S4.pdf]

International Journal of
Environmental Research and Public Health

MDPI

Table S1. Effect of intergroup comparison under high and low strength of comparison. (Using different cutoff values as the grouping criteria).

	Using 1st as the Cutoff Value			Using 2nd as the Cutoff Value		
	Model 1	Model 2	Cross-Model	Model 3	Model 4	Cross-Model
	1st	2nd–25th	Difference	1st–2nd	3rd–25th	Difference
B	11.551	6.870 **	4.681	12.029 *	6.640 **	5.389
SE	(8.000)	(3.076)	(4.948)	(7.101)	(3.122)	(4.198)
N	133	215		136	212	
R^2	0.016	0.023		0.021	0.021	

* $p < 0.10$, ** $p < 0.05$.

Table S2. Effect of intergroup comparison under high and low strength of comparison. (Using different cutoff values as the grouping criteria).

	Using 4th as the Cutoff Value			Using 5th as the Cutoff Value		
	Model 5	Model 6	Cross-Model	Model 7	Model 8	Cross-Model
	1st–4th	5th–25th	Difference	1st–5th	6th–25th	Difference
B	11.779 *	6.379 **	5.400	10.048 *	6.471 *	3.577
SE	(6.115)	(3.202)	(3.584)	(5.289)	(3.313)	(3.428)
N	141	207		148	200	
R^2	0.026	0.019		0.024	0.019	

* $p < 0.10$, ** $p < 0.05$.

Table S3. Moderating effect of prior water usage under different strength of comparison. (Using different cutoff values as the grouping criteria).

	Using 1st as the Cutoff Value			Using 2nd as the Cutoff Value		
	High-Strength	Low-Strength	Reverse-Treated	High-Strength	Low-Strength	Reverse-Treated
B	−0.682	−9.830 ***	−18.487	−5.096	−9.429 **	−18.487
SE	(7.972)	(3.762)	(12.536)	(6.963)	(3.875)	(12.536)
N	133	215	138	136	212	138
R^2	0.241	0.196	0.191	0.237	0.196	0.191

This table only reports results about the effect of the multiplicative term, which indicates whether the moderating effect of prior water usage exists. ** $p < 0.05$, *** $p < 0.01$.

Table S4. Moderating effect of prior water usage under different strength of comparison. (Using different cutoff values as the grouping criteria).

	Using 4th as the Cutoff Value			Using 5th as the Cutoff Value<br>Using 4th as the Cutoff Value<br>Using 5th as the Cutoff Value		
	High-Strength	Low-Strength	Reverse-Treated	High-Strength	Low-Strength	Reverse-Treated
B	−6.011	−9.266 **	−18.487	−4.677	−10.351 **	−18.487
SE	(6.330)	(3.955)	(12.536)	(5.345)	(4.214)	(12.536)
N	141	207	138	148	200	138
R^2	0.239	0.196	0.191	0.245	0.193	0.191

This table only reports results about the effect of the multiplicative term, which indicates whether the moderating effect of prior water usage exists. ** $p < 0.05$.
